# Sand consolidation using enzyme-induced carbonate precipitation: new insights on temperature and particle size effects

**DOI:** 10.1038/s41598-023-42792-w

**Published:** 2023-09-19

**Authors:** Kamal Omarov, Sulaiman A. Alarifi, Mohamed Mahmoud, Muhammad Shahzad Kamal, Mobeen Murtaza, Abdulmohsen Humam, Manar M. AlAhmari

**Affiliations:** 1 Petroleum Engineering Department, King Fahd University of Petroleum and Minerals, 31261 Dhahran, Saudi Arabia; 2 Center for Integrative Petroleum Research, King Fahd University of Petroleum and Minerals, 31261 Dhahran, Saudi Arabia; 3 Saudi Aramco, 31311 Dhahran, Saudi Arabia

**Keywords:** Fossil fuels, Biomineralization

## Abstract

Sand production is a major issue in the oil and gas industry. Unconsolidated sand can be produced with the oil or gas a cause many issues to the production facilities. Enzyme-induced carbonate precipitation (EICP) is a promising method for sand consolidation and is characterized by its environment friendliness. Numerous studies have shown its effectiveness in ambient conditions. However, oil and gas downhole well operations are high pressure and high-temperature conditions. The objective of this study is to investigate effect of high temperature on EICP reaction and its efficiency in terms of uniformity to consolidate different types of sand samples. In this paper, the behavior of EICP solutions is examined in high temperatures from 25 to 90 °C. The study shows that high temperature environment doesn’t handicap efficiency but in contrast it can favor the reaction if optimum concentration of reactants has been selected. The temperature effect is also discussed in terms of controllability of reaction which can favor application of reaction. Qualitive analysis shows when EICP solutions containing more than 50,000 ppm of metal ions and stoichiometrically surplus urea requires exposure to heat for reaction progress. The effect of sand particle size and its implication on the consolidation process was examined. Particle size of fine and medium sand ranged from 125 to 250 µm and 250 to 425 µm respectively while for coarse sand 70% sand particle size was between 425 and 700 µm. Designed EICP solutions achieve 9,000 psi for medium and almost 5,000 psi intrinsic specific energy for coarse sand samples. However, treated samples were subject to non-uniform distribution of strength of which can be up to 8,000 psi difference between top and bottom half of the samples.

## Introduction

Due to environmental concerns, interest to alternatives of ordinary Portland cement (OPC) has risen sharply. One of the promising alternatives is bio-cementation which is further classified as bio-inspired and bio-mediated. Compared to energy-intensive OPC which emits vast quantities of byproducts to air, bio-techniques uses either living organism or mimic the processes to catalyze reaction and yield carbonate ions. Main source of carbonate ions is hydrolyzed urea, and it will precipitate different minerals depending on the metal ions introduced to the reaction as seen in Eqs. 1 to 9^1^:

1 $${\text{H}}_{{2}} {\text{N}} - {\text{CO}} - {\text{NH}}_{{2}} + {\text{2H}}_{{2}} {\text{O}} \to {\text{NH2COOH}} + {\text{NH}}_{{3}}$$

2 $${\text{NH}}_{{2}} {\text{COOH}} + {\text{ H}}_{{2}} {\text{O}} \to {\text{NH}}_{{3}} + {\text{H}}_{{2}} {\text{CO}}_{{3}}$$

3 $${\text{H}}_{{2}} {\text{CO}}_{{3}} \leftrightarrow {\text{HCO}}_{{3}}^{ - } + {\text{H}}^{ + }$$

4 $${\text{2NH}}_{{3}} + {\text{ H}}_{{2}} {\text{O}} \to {\text{2NH}}_{{4}}^{ + } + {\text{2OH}}^{ - }$$

5 $${\text{HCO}}_{{3}}^{ - } + {\text{H}}^{ + } + {\text{2OH}}^{ - } \leftrightarrow {\text{CO}}_{{3}}^{{{2} - }} + {\text{2H}}_{{2}} {\text{O}}$$

6 $${\text{CaCl}}_{{2}} \to {\text{Ca}}^{{{2} + }} + {\text{2Cl}}$$

7 $${\text{MgCl}}_{{2}} \to {\text{Mg}}^{{{2} + }} + {\text{2Cl}}^{ - }$$

8 $${\text{Mg}}^{{{2} + }} + {\text{CO}}_{{3}}^{{{2} - }} \to {\text{MgCO}}_{{3}}$$

9 $${\text{Ca}}^{{{2} + }} + {\text{CO}}_{{3}}^{{{2} - }} \to {\text{CaCO}}_{{3}}$$

Depending on the type of catalyzer introduced to the reaction, bio-cementation techniques will be classified as enzyme induced carbonate precipitation (EICP) or microbially induced carbonate precipitation (MICP). EICP uses different enzyme source such as jack bean meal (JBM), soybean, pigeon pea, cotton seed among which JBM is considered arguably most efficient^2^. Since pure enzyme is vulnerable under different environmental condition (alkalinity and temperature), shielding it with different stabilizers became a common practice^3–5^. The products of hydrolysis, carbamate, and ammonia are unstable in aqueous solution so that they degrade into bicarbonate and ammonium ions. The presence of hydroxide ions will increase the pH and shift bicarbonate to carbonate ions. Therefore, it is desirable to keep high pH by increasing molar ratio of urea to metal ions or improving enzyme functionality and at the same time, high alkalinity so that the reaction becomes less prone to environmental conditions^6^. For calcium carbonate minerals, threshold value is a pH below 7 at which it dissolves in aqueous environment. On the other hand, highly alkaline environment degrades performance of urease, 6.9 pH was optimum value, according to studies of Chouchan et al.^7^.

Carbonate ions will give different precipitates with different forms in the presence of different inputs. Calcium ions in the form of CaCl_2_ are commonly used for the purpose of forming calcium carbonate. These precipitates can appear in amorphous (noncrystaline phase) or in different forms of polymorphs such as calcite, aragonite, vaterite^8^. Rhombohedra calcite is the most stable form at which enzyme induced carbonate mainly yields^9,10^. The presence of trivalent or divalent ions can modify polymorph form or crystal sizes of calcium carbonate. Larsen et al. used Mg^2+^ to Ca^2+^ ratio of 1:1000 or deficit amount of CaCl_2_ with respect to urea to provide larger crystal size^11^. This was further validated by a study by Apriliani et al. where Mg^2+^ ions lead to decreased rate of carbonate precipitation and modified morphology and structure^12^. Also, Boyd et al. observed transition from angular to spherical precipitates with an addition of Mg^2+^ ions^13^. The introduction of Ca^2+^ in different forms is also experimentally analyzed by Sung Sik et al. where they used calcium hydroxide and calcium nitrate as metal ion source. However, CaCl_2_ was validated as most efficient due to higher solubility in water^14^. Since the main aim of reaction is to maximize amount of precipitation so that more minerals coats sand grains together, it is desirable to find an addition of Mg^2+^ so that it increases precipitation yield. Putra et al. showed that replacement up to 20% of 0.5 M Ca^2+^ ions can yield higher amount precipitation. In parallel, formation of aragonite, less stable but more compact polymorph, intensifies leading to improved geomechanical properties^1^. Aragonite is high strength mineral with higher Mohr hardness than calcite and also has capability to improve strength of rubber and plastics as a filler^15,16^. In another study by Putra et al., Mg^2+^ ions are added to reaction in the form of MgSO_4_ to promote not only the presence of aragonite but also gypsum. Substitution up to 0.04 M/L of 1 M/L CaCl_2_ gives rise to aragonite formation, while 0.1 M of MgSO_4_ yield significant amount of gypsum. Although higher amount of MgSO_4_ raised PR even up to full capacity, geomechanical properties does not follow the same trend. Highest unconfined compressive strength (UCS) of 87 psi was achieved by 0.04 M/L of CaCl_2_^17^. These studies showed that introduction of Mg^2+^ ions improved both precipitation and geomechanical properties.

There is a positive correlation between precipitation efficiency and its effect on strength of samples. The main challenge is to achieve a uniform distribution and shield reaction from the negative effects of surrounding environments. It has been experimentally validated that multiple pore volume injection of EICP solution has enhanced consolidation by introducing more carbonate precipitation to contact points of soil particles. Almajed et al. achieved 2 and up to 10 times stronger samples with incremental treatment cycles for Ottawa sand while Martin et al. reported up to 58 psi increment^8,18^. However, injection of excess amount of slurry can increase amount of precipitation in porous media but cannot affect significantly distribution of precipitation. There is wide range of studies of different additives used for this purpose such as on-fat milk powder, sodium alginate, sodium montmorillonite, xanthan gum and glycerol. Almajed et al. concluded that addition of non-fat milk reflects 0.5 MPa strength difference when amount of precipitated amount is the same^3^. Positive effect of sodium alginate, sodium montmorillonite and xanthan gum are also validated experimentally, and their efficiency are thanks to their cross-linking and thermal properties^19,20^. Another crucial point is sensitivity of consolidation to curing time and surrounding moisture. Lee et al. concluded that especially for higher concentration cement slurry more curing time are needed and the time can increase when surrounding moisture content is high^21^. However, contribution by this metal ion is restricted up to some threshold value, overall 1 M of metal ion concentration.

The efficiency of precipitation in most studies is analyzed using precipitation ratio which is defined as the actual mass of precipitant divided by its theoretical mass. In these studies, calcium carbonate is precipitant and theoretical mass will be ascertained from stoichiometric ratios holding the assumptions that all available carbonate ions and calcium source lead to biomineralization. Mathematical expression can be written as (Eqs. 10 and 11):

10 $${\text{Precipitation}}\;{\text{Ratio}}\left( {{\text{PR}}} \right) = \frac{{m_{p} }}{{m_{t} }}$$

11 $$m_{t} = C \cdot V \cdot M$$

where m_p_ is the mass of precipitated material, m_t_ is the theoretical mass, C is the concentration of solution (mol/L), V is the volume of solution and M is the molar mass of precipitant.

Nevertheless, temperature effect on EICP reaction is not studied by many authors. According to studies of Nemati et al. temperature increase from 20 to 50 °C effects positively on the final yield of reaction^5^. The favorable effect is also validated by Manar AlAhmari studies where maximum temperature is raised to even to 140 °C. It has been precipitation process happens after 100 °C regardless of presence of urease^4^. Another study on temperature effect has been done by Feder et al. Different types of catalyzers for urea hydrolysis has been tested and their performance monitored for different temperatures. Results show that temperature increment to 60 °C can stimulate urea hydrolysis while after that point enzymes (jack urease bean) can lose its functionality^2^. Many other additives for sand consolidation are studied in the literature such as different types of polymers and nanoparticles, each additive has its limitations when it comes to application in the oil and gas industry^22,23^.

Despite those studies, there is research gap in detailed analysis of temperature effect on EICP and their performance on consolidation process. In more detail, studies on EICP consolidation are limited to single strength measurement for treated samples. However, if the application is extended from laboratory scale to field scale, uniformity, and precipitation distribution will be one first factor to be considered.

The objective of study is to investigate effect of high temperature (higher than ambient condition) on EICP reaction and its efficiency in terms of uniformity to consolidate different types of sand samples. Their effect will be measured and analyzed through predesigned reaction and consolidation metrics.

## Materials and equipment

### Reactants and catalyzer

To perform temperature effect studies on EICP, reactants which included MgCl_2_*6H_2_O, CaCl_2_*2H_2_O, urea and catalyzer of urease from jack bean has been used. Those chemicals are product from Sigma Aldrich and VWR chemicals with 99% purity.

Enzymes are biopolymeric macromolecules and possess 3-dimensional structures with an ability to catalyze the reactions without being consumed. They achieve faster rate of reactions by lowering activation energy between reactants and transition state. The type of enzyme that has been used in this study is urease enzyme which is commonly occurring protein in higher order plants or microorganisms. Urease can be derived from different sources, but jack-bean urease is one of widely used and studied catalyzer^24^. Type of enzyme used in this study is Type III with 15,000–5,000 units/g solids. Enzymes catalyzer for this reaction is kept in refrigerator. Solubility of chemicals in solvent is validated using at least 10 min curing for each solution.

Urea, also known as carbamide, is an organic compound with chemical formula of CO(NH_2_)_2_. The structure is formed by 2 amino groups and combined by carbonyl functional group. Other characteristics include good solubility in water, neither acidic nor basic and characterized by colorlessness and odorless solid state (powder).

Main source of carbonate ions is from hydrolysis of urea. A faster rate of hydrolysis is achieved by nucleophilic attack of urea carbonyl oxygen to nickel atoms of urease active site^25,26^. The interaction is enhanced by hydrogen bonding between amino groups of urea and Ni atom. Hydrolysis will initiate by resulting release of NH_3_ which weakens urea and urease coordination and finally by breaking of carbamate group NH_2_COO^-^. This unstable group will give rise to formation of CO_2_. A summary of the materials used in the EICP solution and their functions is provided in Table 1.

**Table 1:** EICP solution materials and their functions.

Material	Function
Urea (CO(NH_2_)_2_)	Source of carbonate (CO_3_^2−^)
Jack-bean urease	Enzyme catalyzer
MgCl_2_*6H_2_O	Source of Mg^2+^ (salt)
CaCl_2_*2H_2_O	Source of Ca^2+^ (salt)

### Sand samples

**Figure 1:**
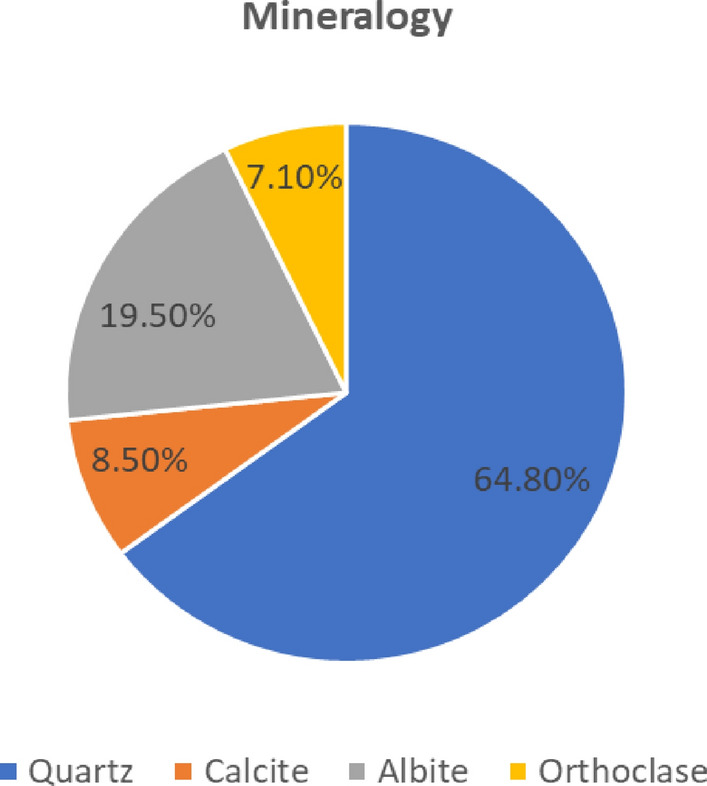
Mineral content (in %) of raw sand.

Prior to the treatment of sand, raw sand samples have been exposed to mineralogical analysis. With regard to the mineralogy (Fig. 1) where calcite and quartz are main constituents, the sand used in this study can be characterized as sandstone. There was also a small trace of clay minerals such as albite. To avoid their negative effect on consolidation, raw sand samples are exposed to water flushing and followed by filtering using vibratory sieve shaker.

### Coupled plasma optical emission spectroscopy

To measure metal ionic concentration of solutions, Inductively Coupled Plasma Optical Emission spectroscopy (Optima 8000 ICP-OES) has been utilized . The working principle of equipment is based on analytical analysis of emitted light from exposed solution. Each type of atom or ions carries unique characteristics of energy absorption and subsequent transition of energy level for their electrons. Energy level transition happens when electrons are exposed to high energy source (argon plasma). When excited electrons bounce back to their ground level, they will emit light with specific wavelength which is proportional to concentration^27^. Since the equipment can detect up to 100 ppm concentration, it has been calibrated with 4 specific calibration fluids of 1, 10, 25 and 50 ppm.

### Sand sieving

Prior to the treatment of sand by EICP solutions, it has been filtered using Retsch Vibratory Sieve Shaker. To achieve desired sand particle distribution, at least 10 min are given for equipment to vibrate the sand sample. Sand are sieved between 125 and 250 µm to achieve for finer ones while for medium sand between 250 and 425 µm sieves has been used. The particle size higher than 425 µm are considered as coarse sample. Three types of sand have been prepared and they are also flushed with water to avoid any contamination. All sand samples are oven dried at 90 °C for at least 4 days.

### Scratcher test

The Wombat scratcher machine was used for the scratch test. This technique of assessing rock mechanical property is considered as quasi nondestructive test. It measures both tangential and normal components of force. Adjustable parameters are relative velocity between cutter and rock, depth of cut and cutting angle.

Based on geomechanical behavior of rock through, it will perform ductile or brittle behavior. Especially for ductile, there is a well-studied correlation between intrinsic specific energy and uniaxial compressive strength. Intrinsic specific energy is required energy to remove a unit volume of rock. This method offers several advantages over conventional methods such minimal sample preparation, non-destructive, reproducible tests, and ability to create log of strength through the sample^28,29^. This will allow for the recording of strength measurement with several data points along the entire length of the consolidated sample.

## Methodology

### Temperature sensitivity analysis

To perform temperature sensitivity analysis, reactant of reaction which are CaCl_2_*2H_2_O, MgCl_2_*6H_2_O and Urea have been prepared following the procedure. First, mix different concentration of CaCl_2_*2H_2_O, MgCl_2_*6H_2_O and Urea using mixer for at least one hour. Then, filter the solution. After that, cure the solutions for at least one day. Lastly, measure ionic concentration using Optima 8000 ICP-OES. The concentration of solutions is in increasing order from M4 to M2 and their concentration can be found in Table 2. For all solutions, molar ratio of urea to metal ions are taken higher than one to ensure favorable condition for precipitation such as high pH and adequate amount of carbonate ions.

**Table 2:** Reactants.

Sample name	Ca^2+^ (ppm)	Mg^2+^ (ppm)	Urea (ppm)
M2	57,087	26,655	180,205
M3	53,000	23,085	150,171
M4	49,457	19,885	120,137

Preparation of reactants has been followed by EICP solution preparation. In contrast to most studies, sensitivity analysis has been performed based on volume ratios. By keeping constant volume of 40 ml, the volume of catalyzer has been shrunk from 20 to 5 ml for each M2, M3, M4 reactants (Table 3). The general procedure starts by preparing 1 g/L solution from jack-bean urease. Then, cure catalyzer for 1 h and filter it. Then, prepare final EICP solutions which contain 1 to 7 volume ratios of reactant to catalyzer an overall constant volume of 40 ml. Lastly, mix them thoroughly.

**Table 3:** EICP solutions.

Sample name	Reactants	Catalyzer	Ca^2+^	Mg^2+^	Urea
	ml	ml	ppm	ppm	ppm
M2V1	20	20	28,544	13,327	90,000
M2V2	30	10	42,816	19,999	135,000
M2V3	35	5	49,952	23,323	157,500
M3V1	20	20	26,500	11,542	75,000
M3V2	30	10	39,750	17,314	112,500
M3V3	35	5	46,375	20,199	131,250
M4V1	20	20	24,728	9,942	60,000
M4V2	30	10	37,092	14,914	90,000
M4V3	35	5	43,275	17,399	105,000

**Figure 2:**
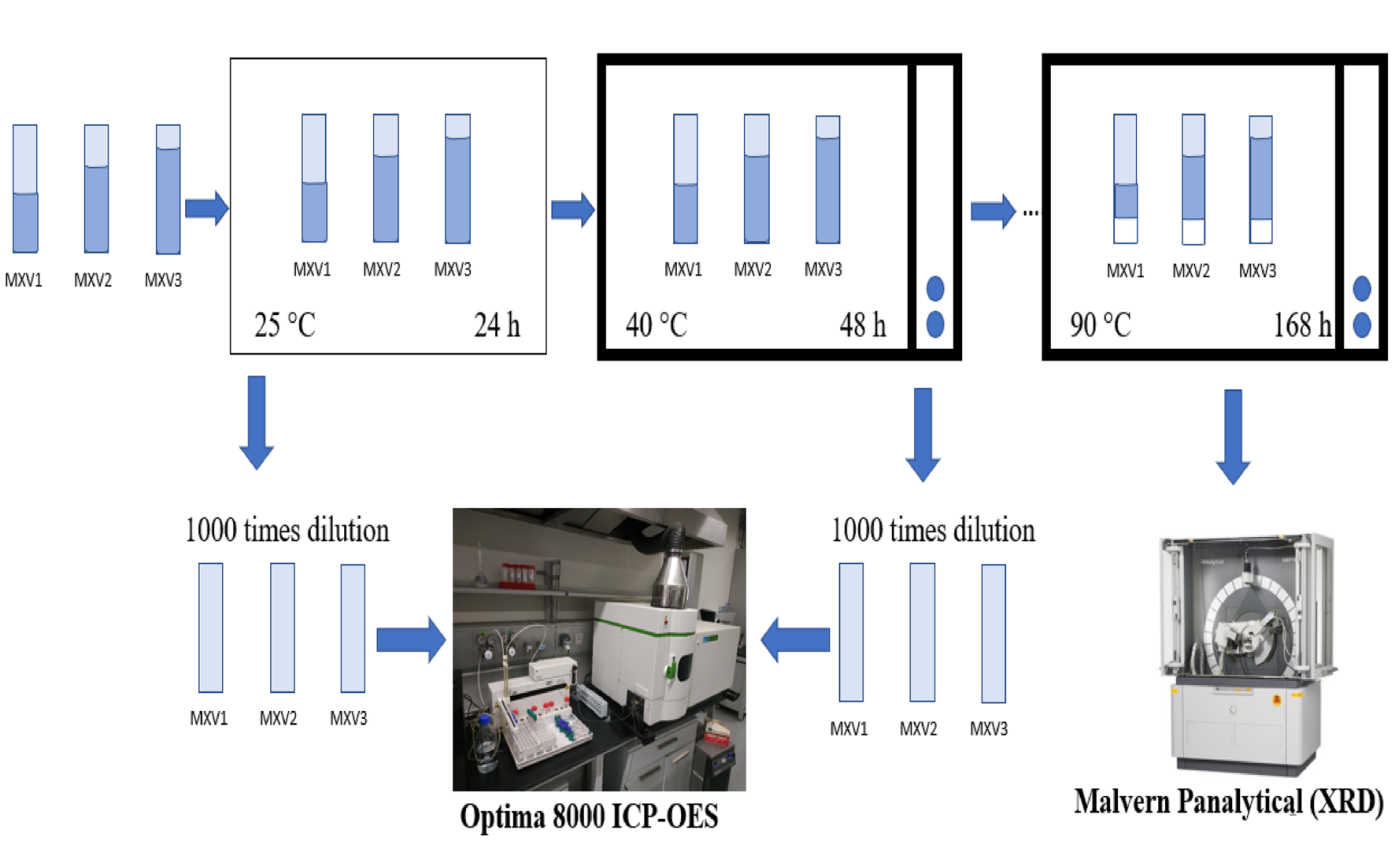
Workflow of temperature studies.

To perform temperature sensitivity analysis, the temperature has ranged from room temperature to 90 °C with an increment of 10 °C. At each temperature increment, a 0.1 ml sample has been taken and diluted 1000 times using deionized water as described in Fig. 2. Those samples are exposed to elemental concentration measurements by Optima ICP-OES. At the final step, induced precipitation has been collected and dried for mineralogical analysis.

### Consolidation studies

#### Sand preparation

Prior to the treatment of sand by EICP solutions, it has been filtered using Retsch Vibratory Sieve Shaker. To achieve desired sand particle distribution, at least 10 min are given for equipment to vibrate the sand sample. Sand are sieved between 125 and 250 µm to achieve for finer ones while for medium sand between 250 and 425 µm sieves has been used. The particle size higher than 425 µm are considered as coarse sample. Three types of sand have been prepared and they are also flushed with water to avoid any contamination. All sand samples are oven dried at 90 °C for at least 4 days.

**Figure 3:**
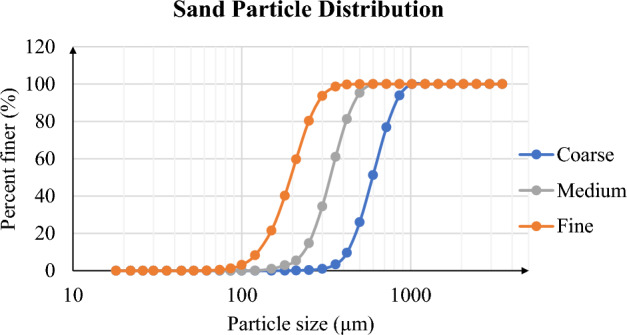
Types of loose sands.

Sand particle size distribution is shown in Fig. 3. It can be concluded that desired distribution has been achieved using sieve shaker with some minor population outside the range. For example, fine sand consists of 3% of particle which is lower than 125 µm while this is 5% of less than 250 µm for medium sand.

#### Consolidation procedure

To perform consolidation experiments, 3 cement solutions are selected from among the solutions in Table 3. Since the main target of study is to analyze sand particle size effect, maximum amount of catalyzer has been taken to ensure adequate amount of precipitation (Table 4).

**Table 4:** Sand treatment solutions.

Sample name	Reactants (ml)	Catalyzer (ml)	Ca^2+^ (ppm)	Mg^2+^ (ppm)
M2V1	20	20	27,446	13,327
M3V1	20	20	26,500	11,542
M4V1	20	20	24,728	9,942

After preparation of loose sand samples, the approximate pore volume of sand samples has been determined. The mass of samples ranged from 162 to 177 g and occupied a cell volume of 40 mm diameter and 60 mm height. All the samples are treated with 40 ml of EICP solution which is higher than pore volume to make sure full contact of sand. Mix-compact treatment method is applied, and samples are cured at least 10 days at 90 °C.

## Results and discussion

### Temperature sensitivity analysis

The progress of EICP reaction depends on concentration of metal ions and source of carbonate ions. Hydrolyzed urea enriches the solution with carbonate ions and when those ions confront with reactive metal ions such Ca^2+^ and Mg^2+^, they give rise calcium carbonate, dolomite or other chemical compounds. Since precipitation process consume metal ion concentration of solution, consumption trend of Ca^2+^ and Mg^2+^ can help to track the progress of reaction. This method is more accurate and advantageous over conventional EICP sensitivity tests which is based on weight of precipitation. Considering the high cost of enzyme catalyzer, most of test-tube studies in literature are conducted in limited volumes (up to 50 ml) that yields little weight of precipitates which is difficult to analyze and choose optimum concentration.

**Figure 4:**
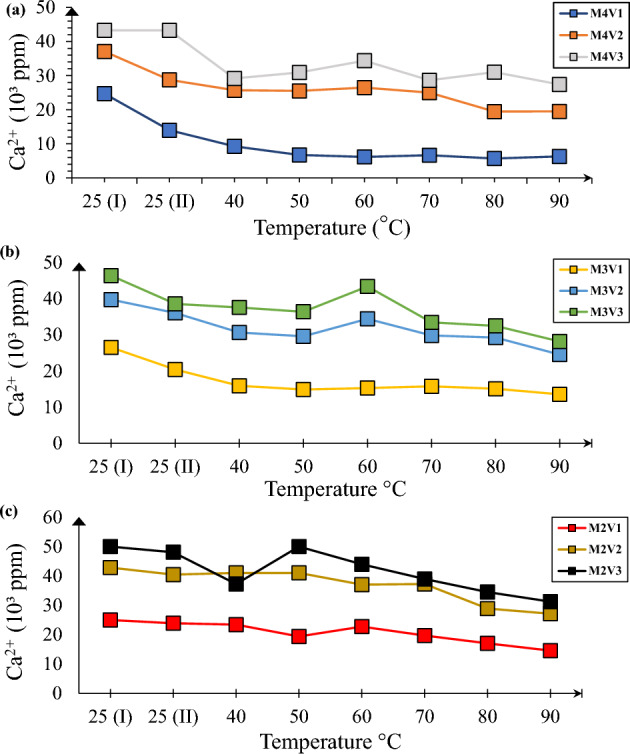
Ca^2+^ consumption dynamics for samples M4 (**a**), M3 (**b**) and M2 (**c**).

It can be observed from Fig. 4 all of the solutions experience uninterrupted downward trend of Ca^2+^ consumption as they are exposed to higher temperatures. Especially for highly saturated solutions M2 in Fig. 4a, there is still potential carbonate precipitation. M2V3 solutions which contain negligible amount of catalyzer volume to reactant exhibit competitive trend against M2V1 which has equal volume ratios of reactant and catalyzer. Regarding EICP solutions from less saturated M3 solution, effect of temperature become less pronounced. For example, M3V1 has achieved equilibria at 50 °C and incremental temperature is no longer effective for precipitation process. However, as in M2 solutions, higher temperature becomes necessity when volume of jack urease bean solution has been shrunk. As can be seen from Fig. 4b, increment after 50 °C leads to fluctuations in metal ion concentration probably due to generation of carbonic acid. However, this followed by equilibria shift to carbonate ions and consumption by metal ions for M3V2 and M3V3. A solution containing comparatively least amount of urea and metal salts shows interesting behavior in terms of efficiency of reaction. M4V1, as described in Fig. 4c, experiences sharpest and fastest drop of calcium concentration reaching a value of below 10,000 ppm.

**Figure 5:**
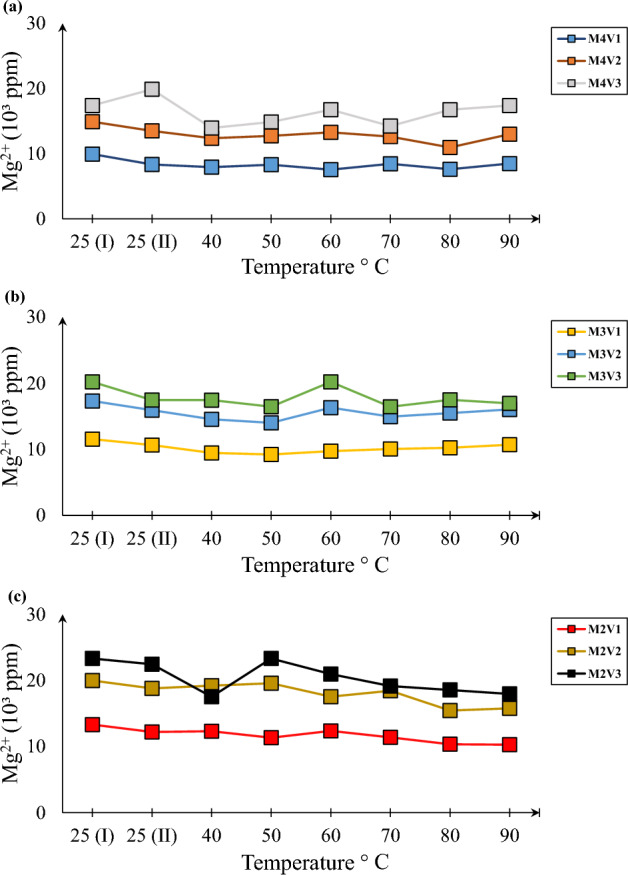
Mg^2+^ consumption dynamics for samples M4 (**a**), M3 (**b**) and M2 (**c**).

Another interesting question is whether Mg^2+^ is effective to get efficient precipitation in terms of mineralogy and mass. In spite of numerous literatures showing positive of effect of Mg^2+^ presence for mass of precipitation^1,30^, they exhibit quite unreactive nature. From Fig. 5a to 5c, it is clearly observed that Mg^2+^ consumption is significantly lower than Ca^2+^. In a high concentration environment such as in M2 and M3, there is lesser chance of contribution to precipitation while some contribution can be expected if solution contains less than those.

It has been experimentally validated that temperature fills the gap of catalyzer for induced carbonate precipitation in contrast to other studies which shows efficiency of enzyme degrades after 70 °C^2^. However, trends in Figs. 4 and 5 show that the reaction progresses up to even 90 °C temperature. This fact can be explained by the nature of the reaction. Since urea hydrolysis is endothermic^31^, continuous supply of heat for more than 7 days stimulates the hydrolysis process and induces precipitation.

**Figure 6:**
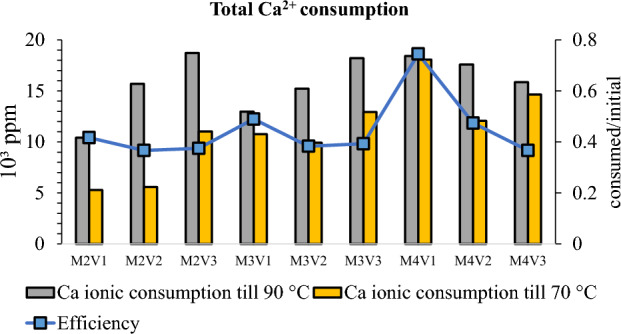
Comparison of metal ion consumption.

Since this progress is earned also at the expense of high reactant concentration, efficiency of reaction is another factor to consider. Figure 6 illustrates, total change of Ca^2+^ concentration and estimated efficiency based on consumed and introduced reactants. For combinations of M2 and M3, solutions containing more reactant to catalyzer induce more Ca^2+^ consumption. In terms of efficiency, nearly half of existing ions lead to precipitation of carbonate minerals. Nevertheless, there is a clear sign of enhancement for M4V1 solution where almost 80% of existing Ca^2+^ ions converted to precipitates. The faster and efficient reaction rate can be attributed better enzyme performance at low metal concentration^6^.

**Figure 7:**
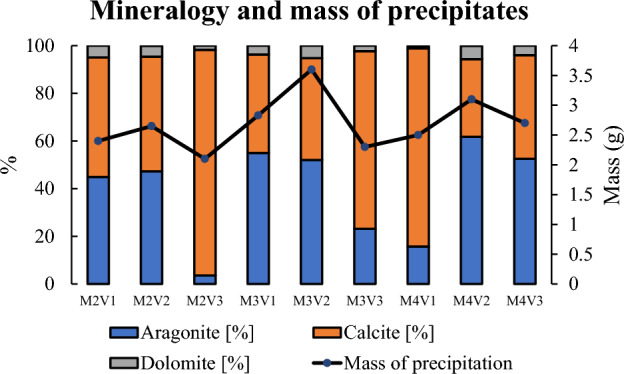
Mineralogy and mass of precipitates.

Since contribution of Mg^2+^ to precipitation is negligible according to ionic studies, the question arises on whether it has any effect on mineralogy. Figure 7 illustrates minerals comprising precipitation collected from test tubes. Percentage of dolomite mineral that is almost below 5% further confirms that Mg^2+^ is less competent to Ca^2+^ to induce carbonate precipitation in high concentration environment. On the other hand, minerals are not only characterized based on their chemical formula but also their crystal structure. For all solutions, a minimum of 40% of calcium carbonate is in the form aragonite morphology while the rest possesses calcite structure. From the aspect of soil improvement, it is debatable which one of them is more efficient.

Figure 7 also highlights the mass of precipitates. It fluctuates between 2.5 and 3.5 g. Solutions categorized by M2 and M4 show similar trends and weight of mass. Nevertheless, the mass of M3V2 which is almost 1 g higher than average mass of all is worthy to consider. This reaction product contains 50% less catalyzer than its closest counterpart and highlights how high temperature environment can stimulate reaction in the lack of catalyzer.

Deficit volume of enzyme is not only effective in terms of cost but also can be effective in terms of application. For ambient temperature, rate of enzyme induced precipitation is a function of strength of catalyzer and concentration. From the aspect of application which is not limited to fracture plugging and lost circulation mitigation, EICP cement need significant amount of time to imbibe through pore space and once stabilized to contact with formation^5,32^Click or tap here to enter text.. When the reaction is too fast, precipitates will clog the pore space and will handicap further movement of fluid to the contact points of soil particles. This scenario is most probable for low reactant concentrations. The solutions containing 50% percent of catalyzer, especially M4V1, give rise to significant precipitation in short time. However, when catalyzer volume shrinks to 25% and even less 12.5%, the temperature and timing effect dominates. Those solutions can be easily delivered to the area of interest and precipitation can be induced by exposing them to higher temperature in comparatively longer period of time. Table 5 summarizes the visual inspection of EICP occurrence of all the samples at different temperatures from 25 to 90 °C.

**Table 5:** Visual inspection of EICP occurrence.

Qualitive analysis	25 °C	40 °C	50 °C	60 °C	70 °C	80 °C	90 °C
M2V1	✓	✓	✓	✓	✓	✓	✓
M2V2	×	×	✓	✓	✓	✓	✓
M2V3	×	×	✓	✓	✓	✓	✓
M3V1	✓	✓	✓	✓	✓	✓	✓
M3V2	×	×	✓	✓	✓	✓	✓
M3V3	×	×	✓	✓	✓	✓	✓
M4V1	✓	✓	✓	✓	✓	✓	✓
M4V2	✓	✓	✓	✓	✓	✓	✓
M4V3	×	×	✓	✓	✓	✓	✓

### Sand Consolidation Using EICP.

The second part of the study highlights how effective the EICP solutions are to consolidate different types of sand. To measure strength distribution, the sample has been exposed to scratch test and at the end of each, high resolution camera is used to take picture of scratched samples. Prior to distribution analysis of both strength and precipitation, it is worthy to understand how treatment methodology can affect the results. Sands treated with injection or surface percolation of EICP solutions are most vulnerable ones to non-uniformity^8,33,34^. Mix-and-compact method has been preferred over other considering their tendency of generating better contact between cement and treated sand.

**Figure 8:**
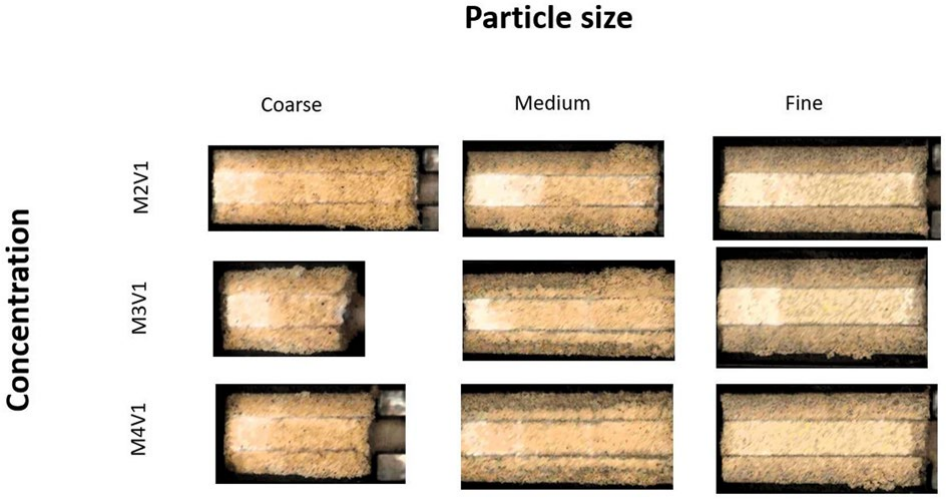
Visual inspection of treated sand samples.

The study contains 9 consolidated samples which are combinations of different sand particle sizes and concentration. From visual inspection and intensity of colors in Fig. 8, it can be observed that from lower to higher concentration, there is more precipitation in pore space. However, those pictures not only help to detect them but also to see how they are distributed along the samples. From fine to coarse sand, spatial distribution of carbonate minerals differs. Especially, for fine sand, uniform distribution of precipitates is observed. Another essential fact is that in spite of the same curing and treatment methodology, coarse sand has cracked from the higher half part. It can be explained by non-uniform consolidation and nature of big sand particles which requires bigger precipitates to bind them together.

**Figure 9:**
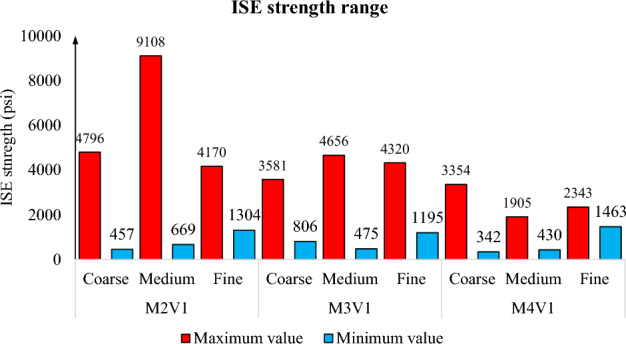
Basic statistical analysis of strength.

Another factor to address is how amount and degree of uniformity of precipitation affects the strength of samples. Figure 9 generalizes this phenomenon for 3 different sand types. Minimum and maximum ISE strength are reflected on the figure to highlight the importance of strength distribution. Generally, it can be observed that the difference can be up to 4,000 psi for coarse sand and this value can rocket to 8,500 psi if particle size is around 250 and 475 μm. On the other hand, fine sand is showing more stable consolidation with a maximum of 3,000 psi for high concentration and less than 1,000 psi when concentration is further reduced. In comparison to other studies, ISE strength values are higher due to absence of fines which has negative effect on consolidation^35,36^.

**Figure 10:**
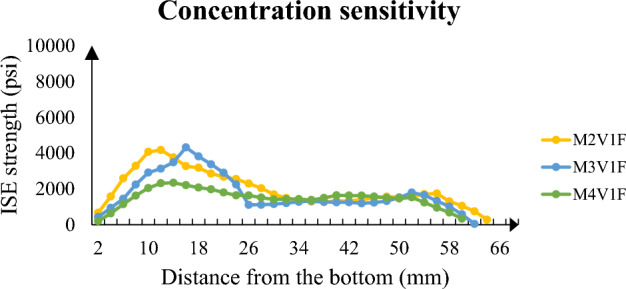
Longitudinal strength distribution of high concentration samples.

**Figure 11:**
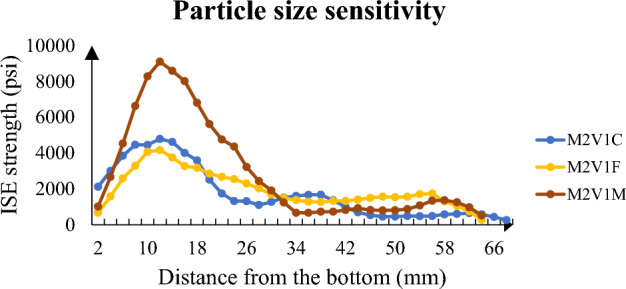
Longitudinal strength distribution of fine sand.

To emphasize concentration and particle size, Figs. 10 and 11 highlight the full distribution of ISE strength of samples. The former illustrates fine sand sample and its magnitude of strength with respect to concentration. In spite of difference in the yield and efficiency of solutions, all give rise to a strength nearly equal at top half of sample and proportional values to concentration at bottom half. The main point here is consolidation uniformity and consolidation potential of sample containing 125 and 250 μm sand particles. It is the range of value which yields uniform consolidation thanks to clogged connected porosity but limited strength up to 4,000 psi.

Figure 11 analyzes consolidation from the aspect of particle size. Highest concentration solutions are analyzed to clearly see the distribution of precipitation. It stands out from graph that when particle size becomes higher than 250 μm, probability of non-uniform distribution rise significantly. Maximum strength has jumped from 4,170 to nearly 9,000 psi. However, such high value of strength cannot be considered efficient because at least 10 times lower value at the top half of sample. Such lower values are also experienced in the top half of coarse sample, but they have twofold less strength at bottom. The difference between coarse and medium sand size can be explained by the limited contact area of coarse sample which has particle diameter with at least 425 μm.

One of the alternatives to address non-uniformity problem is to modify EICP content. Since most of proposed EICP solutions are water-based and drained off as they are injected to the area of interest. Therefore, additives such as biopolymers are introduced to improve viscosity and fluid retention abilities without harming efficiency of reaction. Furthermore, it is important to note that even though the proposed EICP solution provided very high strength consolidated samples, for it be an effective solution to the industry, it has to provide flowability (high permeability) to allow for the oil or gas to flow through this consolidated sand and therefore not harm the well’s productivity. This aspect has not been studied in this work but is considered for future work.

## Conclusion

From the discussion of temperature sensitivity and consolidation, the following conclusions can be drawn:

- For highly saturated EICP solutions, higher temperature environment (up to 90 °C) can favor reaction. It is shown by ICP analysis that solutions which contain higher than 50,000 ppm of Ca^2+^, Mg^2+^ metal ions, and 110,000 urea are especially characterized by sensitiveness to high temperature environments.
- In terms of efficiency, 35,000 ppm of metal ions is threshold value to achieve nearly complete 80% conversion to precipitation. Higher than this value the conversion is limited to 50% but favored with controllability which can positively affect application of EICP solution.
- Despite numerous studies pinpointing Mg^2+^ positive effect on precipitation, for highly saturated solution, they performed less reactive nature. However, their presence contributes to generation of aragonite which can be debatable in terms applicability in consolidation process.
- From visual inspection of scratched sand sample, most of the treated samples exhibit non-uniform distribution of precipitation. Fine sand of particle size between 125 and 250 µm exhibits comparatively more uniformity.
- The relationship between mass of precipitation and ISE strength is strongly non-linear and function of particle size. Medium sand size experiences 2 times increase in ISE strength with an increment of 1.1% of cumulative Ca^2+^ and Mg^2+^ concentration.

## Data Availability

The datasets used and/or analyzed during the current study available from the corresponding author on reasonable request.
